# Text Book of Practical Anatomy

**Published:** 1849

**Authors:** 


### ARTICLE VI.

*A Text Book of Practical Anatomy.* By ROBERT HARRISON, M. D., M. R. I. A., F. R. C. S. I., &c., with additions by an American Physician; with numerous illustrations. pp. 720. N. York, S. S. & W. Wood, 1848; (from the publishers, by express.)

This is the new edition of the Dublin Dissector, enlarged by additions "under the supervision of Robert Watts, jr., M. D., Prof. of Anatomy in the College of Physicians and Surgeons of the University of the State of New York." The idea of making a text book of anatomy, suited for a dissector by being so arranged that the parts can be examined in turn as they are treated of in the work, occurred to us long ago as the thing that was wanted. We see that some of our cotemporaries speak slightingly of the work of changing a Dissector to be a system of anatomy. Ours be it to praise the effort. The dissector or system of *practical* anatomy is the work to make anatomists. The study of anatomy is a dry business in any other than the *practical* way; but thus, well may the student fancy he is repeating the "hymn of praise to the Almighty." Although we have many excellent text books on anatomy already, we cannot say there is not room for this. Not having examined the work in its details, we cannot speak of the additions that have been made; but Prof. Watts' reputation as an anatomist, is a guarantee that they are valuable.

